# Redefining the Role of Nutrition in Infant Food Allergy Prevention: A Narrative Review

**DOI:** 10.3390/nu16060838

**Published:** 2024-03-14

**Authors:** Michael Brandwein, Roni Enten Vissoker, Helen Jackson, Tavierney Rogan, Jana Pitcock, Esther Krinkin, Carina Venter

**Affiliations:** 1 MyOr Diagnostics Ltd., Zichron Yaakov 3093674, Israel; ronientenvissoker@gmail.com (R.E.V.); helen@myorcare.com (H.J.); tavierney@myorcare.com (T.R.); jana@myorcare.com (J.P.); esther@myorcare.com (E.K.); 2 Section of Allergy and Immunology, Children’s Hospital Colorado, University of Colorado, Aurora, CO 80045, USA

**Keywords:** food allergy, prevention, nutrition

## Abstract

Pediatric food allergy remains commonplace, despite the advancement in our understanding of risk factors and prevention modalities for the condition. Early allergen introduction, a dietary intervention, has been endorsed by professional societies globally as an effective primary preventive measure, yet awareness among medical professionals and parents is lacking. Alongside food allergen introduction, overall nutrition, such as diet diversity, also plays an important role in allergy prevention. To address both food allergen introduction and overall nutrition, dietitians play a pivotal role in the dissemination and education of current guidelines to caregivers. This review addresses the particular role of the dietitian in food allergy prevention consultations, providing up-to-date information on food allergies, their development and prevalence, risk factors, dietary factors and an overview of the current guidelines in the United States. This has not been addressed in any of the current food allergy or nutrition guidelines.

## 1. Introduction

Food allergy is a disabling chronic condition, with a staggering 300% rise in prevalence in the past two decades, especially in children [1,2]. In the United States (US), the rise in food allergy sends a child to the emergency room every 3 min [3]. A growing health concern characterized by reproducible adverse reactions upon exposure to specific food antigens, food allergy is characterized by an immunoglobulin E (IgE)-mediated immune response. Symptoms range from discomfort, itchy rashes, nausea, dizziness, and stomach pain to life threatening breathing difficulties, anaphylaxis, and death. This condition can become fatal in minutes, posing constant risk [4,5]. Food-allergy-related psychological distress, bullying, anxiety, feeding dysfunction, and depression is immense [6,7]. The “food allergy tax” costs US families $4000, amounting to $25 billion annually—a 258% increase in households caring for children with food allergy compared to those without [8]. Although some food allergies tend to resolve by early childhood, tree nut and peanut allergies often continue into adulthood [9,10]. Food allergy is further exacerbated by the clustering of atopic conditions, enabling the progression of atopic dermatitis, allergic rhinitis and asthma in a sequential manner, starting early in life. There is clear evidence that having one of the conditions puts one at risk for the others [11]. Children are particularly vulnerable, as 50% of food allergy cases are diagnosed between 0–1 and 85% within the first five years of life, depicting the urgency of addressing and managing food allergy early on.

The widespread prevalence of food allergy, coupled with the burdensome health and social impact of the condition, has prompted the need to discover primary prevention modalities. Research in the past few decades has explored several different approaches, including probiotic supplementation, Vitamin D supplementation, allergen introduction and prophylactic application of emollients, breastfeeding, and others [12]. Early allergen introduction commensurate with complimentary feeding has emerged as a leading prevention modality and has been endorsed by professional societies globally. However, the implementation of these guidelines is lacking, partially due to the lack of a receptive professional audience to educate patients and parents of their existence.

Standard post-natal healthcare interactions are mostly limited to well-baby visits at a pediatrician or general practitioner, and food allergy prevention has generally not been emphasized within the context of these interactions.

Given that the leading evidence-based prevention modality involves a professionally endorsed dietary intervention, we propose that the burden of food allergy prevention can and should be shifted to registered dietitians, trained in the intricacies of food allergy risk, complementary food introduction, and early allergen introduction.

A significant portion of a dietitian’s formal education includes the effective marriage of prevention education and nutrition science, which aligns perfectly with the desire to translate current food allergy prevention guidelines into population-wide reduction in food allergy prevalence. Any signs of a food allergy reaction should trigger an immediate referral to a pediatrician, general physician, or allergist. Dietitians play a crucial role in providing information in food allergy prevention [13], but there is a lack of information about the particular role of dietitians in allergy prevention and which knowledge is required for dietitians to play this important role.

This novel review provides the requisite scientific background for dietitians to successfully guide parents and children through the recent developments in food allergy prevention, thereby empowering parents and dietitians alike in this important endeavor. The review begins with a general overview of food allergies, including development, symptoms, prevalence, and common comorbidities, continues to discuss individual risk factors and how they have been theorized to come together to cause food allergies, and ends with a general overview of infant nutrition guidelines and requirements, with an emphasis on current dietary guidelines to prevent food allergies.

## 2. Food Allergy

### 2.1. Development

Food allergy development typically involves a series of stages, from initial exposure to full-blown development of allergic reactions. The process begins with the first exposure to a specific food allergen, often during infancy. Exposure can occur through various routes, including epicutaneously, through the airway epithelium or through the gastrointestinal tract following allergen consumption. Importantly, non-food related sensitization occurs through these routes of exposure as well. The immune system recognizes the allergenic proteins in food and produces specific antibodies, called immunoglobulin E (IgE), against these proteins. These antibodies bind to mast cells and basophils. With infrequent consumption or repetitive cutaneous or epithelial exposures to the allergen, the immune system can develop a heightened allergic response to the specific allergen, termed sensitization. The exact timing, dosage, and route of exposure that leads to sensitization, as opposed to tolerance, has not been fully characterized. Upon subsequent exposure to allergenic food, the allergen-specific IgE antibodies trigger the release of various chemicals, including histamine, from immune cells. These chemicals cause allergic symptoms, ranging from mild (itching and hives) to severe (difficulty breathing and anaphylaxis). Symptoms may not always be consistent, and can intensify without warning, causing more severe and rapid allergic reactions [14], including anaphylaxis. Anaphylaxis can cause a range of severe symptoms, including difficulty breathing, a drop in blood pressure, loss of consciousness, and potentially death, if not treated promptly with epinephrine [15]. Additionally, increased sialylation, a glycosylation process involving the addition of sialic acid on IgE, has been associated with enhanced allergic reactions, including severe anaphylaxis. The removal of sialic acid from IgE reduces its ability to trigger an allergic response, pointing to an important mechanistic element of FA [16].

### 2.2. Allergenic Foods

There are more than 160 known food allergies to different foods, with prevalence rates differing by specific food and populations affected [17,18,19]. The most common food allergens known to trigger allergic reactions include peanuts, tree nuts like almonds, walnuts, pecans, hazel nuts, macadamia, Brazil nuts, pistachio and cashews, milk and other milk/dairy products; eggs (both whites and yolks); soy-based products; wheat; certain types of fish such as salmon and tuna; and shellfish, including crustaceans (shrimp, lobster, crab), and sesame. Egg and milk allergy often develop earlier on in infancy and have a tendency to resolve over time, whereas peanut and tree nut allergies more commonly develop later on in infancy, usually do not resolve and are more prone to inducing severe allergic reactions [20].

In the U.S., the Food and Drug Administration (FDA) mandates the labeling of nine allergenic foods and/or food groups: peanut, milk, crustacean shellfish, tree nuts, egg, soy, fin fish, wheat, recently sesame. Sesame is an allergen of growing concern, and was recently estimated to affect 0.23% of US children and adults [21,22]. Previous studies also suggests that sesame allergy often persists past childhood and often triggers severe reactions, including anaphylaxis [23,24].

### 2.3. Prevalence of Food Allergies in the United States

The factors associated with the development of food allergy are multifactorial, and likely result from a complex interaction of factors including genetics, diet, and environment. In Western countries, food allergy affects about 8% of children and appears to be rising in other parts of the world, including in urban areas of Asia and Africa [22,25,26,27]. In the United States, 7.6% of children and 10.8% of adults have been found to have probable food allergy [22]. It is important to recognize that the results of research on prevalence of food allergies vary in their inclusion and diagnostic criteria. The U.S. numbers above are largely based upon two large cross-sectional population-based surveys in which reported food allergies were considered IgE-mediated if the reported symptoms to specific allergens met well-defined criteria consistent with IgE-mediated reactions. Specific IgE (sIgE) or skin prick tests are frequently utilized in population studies despite their association with higher rates of false positives, since diagnosis based on oral food challenges (the gold standard) are insufficiently performed due to cost and risk [28]. Studies may also base numbers on data from other clinical reports and testing, including self-reported clinical history of food allergy, clinical or hospital visits for food allergy, or determination of allergen-specific IgE (sIgE) either by skin prick test or by serum sIgE [22]. A small number of studies base food allergy prevalence on OFC outcomes. While food allergies are primarily IgE-mediated, mixed IgE- and cell-mediated and non-IgE-mediated food allergies also exist.

### 2.4. The Atopic March

Atopic dermatitis (AD), allergic rhinitis, and asthma are common food allergy comorbidities, and can often appear as a cluster of diseases or in a sequential manner, a finding which is often referred to as the atopic march. Originally defined over 15 years ago, the atopic march is characterized by a typical sequence of progression of atopic conditions, some of which may become more prominent while others subside [29]. More recent evidence suggests the association of atopic diseases can also be driven by a systemic TH2-dominant immune response to cutaneous inflammation. This progression typically begins in infancy with the development of atopic dermatitis, and continues to the development of other allergic diseases, such as food allergies, allergic rhinitis (hay fever), and asthma. Some birth or early childhood cohort studies suggest that the atopic march only occurs in approximately 50% of children with AD [30,31,32,33,34]. Recently, several biologics targeting the TH2 pathway, including dupiliumab and omalizumab, have been approved to treat conditions associated with the atopic march, and it has been hypothesized that these drugs may help to interrupt the atopic march [35,36].

## 3. Risk Factors for Food Allergy

Understanding the multifaceted factors contributing to the rise in food allergies is crucial for the development of effective prevention strategies, the improvement of early detection, and for fostering research on potential treatments. The following are the most common documented risk factors for food allergy.

### 3.1. Family History of Atopic Conditions

Familial predisposition plays a key role in the likelihood of allergic responses. Individuals with a family history (i.e., parents and siblings) of allergic disease have a higher risk of developing food allergies, and this risk increases with increasing numbers of family members with atopy [22,37,38,39]. A small number of genome-wide association studies have identified specific peanut-allergy associated-loci, yet current guidelines stress that these findings do not support the notion of increased risk when a child has an older sibling with a food allergy [40,41,42].

### 3.2. Epicutaneous Sensitization

The skin is the body’s largest and primary protective organ, serving as a first-order physical barrier against the outside environment [43]. A growing body of literature highlights the critical role of the skin’s epithelial barrier in the rise of both food allergy prevalence and allergic conditions. While it was previously thought that the primary source of antigen exposure was early dietary consumption of allergens (via the GI tract), findings from recent studies have shown that early life exposure to antigens through the skin may be a chief contributor to the development of allergy. Such findings have led to the development of the dual allergen exposure hypothesis, which purports that while high dose oral exposure to food antigens leads to tolerance, low dose cutaneous exposure leads to sensitization.

In fact, the role of an impaired skin barrier, characteristic of atopic dermatitis, has long been associated with the development of allergy [44]. Functional mutations in the Filaggrin protein (FLG) gene also play a key role in epidermal integrity. FLG is an essential protein in the epidermal structure, and plays a key role in creating a strong barrier. In addition, filaggrin protein processing also leads to production of molecules that are part of the skin’s “natural moisturizing factor”, which helps maintain skin hydration and pH, another important aspect of the barrier. Loss of function mutations in the FLG gene are associated both with AD and xerosis (dry skin) [45].

Early life exposure to antigens through a compromised and inflamed epithelial barrier, typical of atopic dermatitis, may be a chief contributor to the development of allergy [46]. About one-third of children with AD are likely to be diagnosed with IgE-mediated food allergy, and that has a relationship with early onset and severe AD [47].

### 3.3. Birth/Mode of Delivery

The vaginal microbiota provides a diverse array of microorganisms that are responsible for preparing the immune system in newborns and infants [48] and vaginal deliveries have been associated with a lower risk for allergic disease compared with delivery of infants via cesarean section. A Finnish study which explored delivery mode of 5552 children from the Finnish Medical Birth Register and their corresponding allergy tests (skin prick tests, specific IgE and open food challenges, OFCs) found that at age 12, the overall incidence of atopic sensitization was 15% for those born by normal vaginal delivery (VD), 20% by assisted VD, 20% by elective CS, and 20% by others, for example emergency CS. Among the children of women without atopic diseases, the incidence of food allergy was 6% for those born by elective CS and 2% for those born by normal VD, while respective incidences were 5% and 6% among the offspring of mothers with atopic diseases [49]. A separate Australian study did not show an association between CS and food allergy, yet a recent systematic review and meta-analysis concluded that CS was associated with an increased risk of food and cow’s milk allergies in children aged 0–3 years [50,51].

### 3.4. Environment

The increasing urbanization and industrialization of the environment also contributes to food allergy risk. The rise in exposure to air pollutants causes an increase in cytokine-induced inflammation and a subsequent decrease in epithelial integrity of the skin, gastrointestinal, and respiratory tract, which are all part of the first barrier of defense and immune responses. These effects are common both in urban dwellers and those who live in a household with an active smoker. Disruption of the epithelial barrier may also result from chemicals in detergents, which skew the immune system towards the Th2 response typical of allergic conditions [52,53]. The geographic movement of populations, causing populations to be exposed to new allergens, may also play a role in increasing FA incidence [54,55].

### 3.5. Vitamin D and Season of Birth

Specific nutrients may also play a role in food allergy prevention in infants. Vitamin D is recognized to be integral to innate and adaptive immunity [56], though randomized, controlled trials (RCT) are sparse, and insufficiency has been linked with increased risk [57,58,59]. Some studies have associated lower exposure to sunlight with food allergy development, while other research has found that higher levels of vitamin D could raise the likelihood of allergic sensitization and food allergy. Other observational studies have highlighted relationships between deficiency of Vitamin D and anaphylaxis. In addition, the NHANES study of more than 3000 children and adolescents found an association between Vitamin D deficiency and higher specific IgE levels, and thereby sensitization to both environmental and food allergens, in children and adolescents, although the relationship was not confirmed [56]. Despite the observed inverse relationship between Vitamin D and food allergy development, several randomized controlled trials have failed to show a reduction in food allergy development when supplementing Vitamin D while breastfeeding or in infancy [60,61,62].

### 3.6. Epithelial Barrier Hypothesis

Epithelial sensitization is compounded by a variety of factors which contribute to the degradation of normal skin integrity. According to the epithelial barrier hypothesis, exposure to the modern urban environment promotes inflammation through the activation of epithelial cells cytokines (interleukin (IL)-25 and IL-33) and slow damage to mucosal barriers [46]. Skin inflammation and the impaired barrier, characteristic of clinical atopic dermatitis and xerosis, consequently makes the skin vulnerable to the entry of food particles that trigger sensitization, inflammation, and allergy. Several RCT’s have attempted to restore skin barrier integrity as a potential primary prevention modality for atopic dermatitis, yet consistent positive results have so far been elusive (reviewed in [63]).

Some of the key environmental factors affecting epithelial health include extreme weather, air pollution, and exposure to pathogens and particulate matter in the environment [44]. One 25-year longitudinal study from France found an increase in food allergy with tree pollen sensitization [64]; indoor molds, air pollutants, and pollen counts have also been correlated with AD flare ups [65]. Skin barrier impairment can also be triggered by pollutants, including microplastics, alkaline soaps and detergents. This decreased barrier function may also occur in the nasal epithelium, allowing for transepithelial allergen passage, and subsequent sensitization and mast cell degranulation.

### 3.7. The Microbiome and Antibiotic Usage

Exposure to a diverse microbiome in early life has a key impact on immunity, and may attenuate food allergy risk by encouraging immune tolerance upon repeat exposure to food antigens [66]. Research clearly underscores the importance of the gut microbiome in immune development early in life. There is also some evidence that the early gut microbiome mediates the association between urban industrialization and increases in allergic disease prevalence [67]. However, development of food allergy may be associated with a less mature microbiome [68], a low microbiota-for-age Z-score during the first year of life [69], lower levels of fecal butyrate, and low levels of propionate [70]. Several trials examining probiotic supplementation to pregnant and/or lactating women or infants failed to demonstrate a significant protective effect and a recent review concluded that probiotic supplementation has little to no effect on food allergy [71].

Antibiotic use in early life has been linked to disruptions in the microbiome and is also a known risk factor for food allergy [66]. A large longitudinal study which examined the data of 30,060 children found that children with three or more courses of antibiotics had greater odds of developing milk allergy, non-milk food allergy, and other allergies compared to children with no antibiotic orders. Separately, prenatal antibiotic prescriptions were shown to be the most important risk factor in a food allergy risk prediction model [72].

### 3.8. Piecing Together the Puzzle

**Figure 1:**
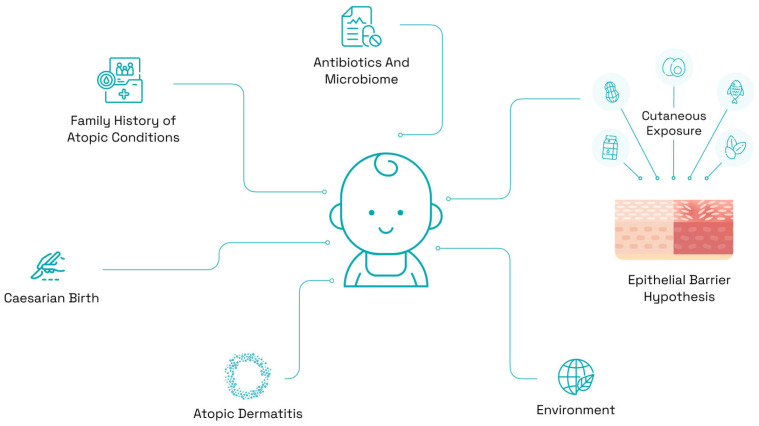
Risk factors for the development of food allergies include family history of atopic conditions, the use of antibiotics and their effect on the microbiome, cutaneous exposure to allergens, the epithelial barrier hypothesis, various environmental influences, a previous diagnosis of atopic dermatitis, and caesarian birth.

The individual risk factors detailed above are diverse, and many are encapsulated in either the dual allergen exposure hypothesis, the epithelial barrier hypothesis, or the skin biodiversity hypothesis. In addition, several maternal and infant dietary factors have been implicated in the development of food allergy (Figure 1). Until recently, actionable insights as to one’s risk were elusive and primarily limited to clinical trials. Our group and others have recently published machine learning approaches to stratify risk based on easily accessible risk factors, paving the way for risk prediction in the clinical setting [73]. Algorithms can be incorporated into a healthcare system’s electronic health record and run in the background or made accessible to parents via a parent-facing digital survey. Once identified, at-risk infants can be targeted for educational interventions to increase the likelihood of incorporating prevention guidelines, as discussed below.

## 4. Dietary Factors in Allergic Disease

### 4.1. Maternal Diet in Pregnancy

The maternal diet is a main component of the infant’s early exposome, and the prenatal maternal diet can contribute to or help prevent the development of food allergy. While the maternal diet has been hypothesized to potentially modulate an infant’s allergic risk, either increasing or reducing the risk of atopy in general (and food allergy specifically) during both pregnancy and lactation, the literature on this subject is sparse.

#### 4.1.1. Food

Consumption of specific foods during pregnancy has been linked to the development of atopic conditions [74], yet these studies have failed to establish such a link to food allergy development. Greater maternal consumption of processed foods during pregnancy has been correlated with increased food allergy in infants [75,76]. Food allergen avoidance during pregnancy has not been associated with reduced food allergy outcomes following birth, may lead to nutritional deficiencies in the pregnant woman, and is not recommended in any of the US guidelines [77,78].

Research from a Colorado pre-birth cohort of mother/offspring dyads was the first study to show associations between an index of maternal dietary intake during pregnancy and multiple offspring allergic diseases. The study used an index that included weighted measures of maternal intake of vegetables, yogurt, fried potatoes, rice/grains, red meats, pure fruit juice, and cold cereals. Vegetables and yogurt were associated with the prevention of any allergy. Adjusted models found a one-unit increase in the index was significantly associated with reduced odds of offspring allergic rhinitis, atopic dermatitis, asthma, and wheeze, but not food allergy [79]. A separate recent study demonstrated a statistically significant association between higher diet diversity in pregnancy and decreased risk of food allergy. The study indicated a 43% decreased risk of food allergy for children whose mothers reported eating the most diverse diet while pregnant compared to children whose mothers reported the eating the most least diverse diet while pregnant [80]. Other diet indices, including the healthy eating index, the diet inflammatory index, and the Mediterranean diet index have not been associated with offspring food allergy development [81,82]. A systematic review from the European Academy of Allergy and Clinical Immunology found limited associations between any diet pattern or index during pregnancy and allergy outcomes in infant and children, including the Healthy Eating Index (HEI), the dietary inflammatory index (DII), and the Mediterranean diet score [83].

#### 4.1.2. Supplements

Fish oil supplementation, a source of omega 3 rich EPA and DHA during early pregnancy can also help reduce sensitization to antigens in infants [84], with EPA potentially altering the balance of T cells away from atopy. One systematic review and meta-analysis found that fish oil supplementation might reduce risk of offspring egg sensitization, although the overall effect on preventing food allergy is not conclusive [85]. More recently, a separate systematic review and meta-analysis concluded that supplementation was not associated with reduced offspring allergy outcomes, and another one reported that it significantly reduced peanut and egg sensitization [83,86].

Increased intake of copper and vitamin C in the prenatal period may protect against food allergy, yet the evidence to support this is weak and pending conclusive results from properly designed randomized controlled trials [83]. Despite the observed observation between Vitamin D levels and food allergy development, Vitamin D supplementation has failed to potentiate as a food allergy prevention strategy is not associated with offspring food allergy [87].

As with the skin microbiome, supplementation of probiotic bacteria in the prenatal period may also help shift the TH1/TH2 balance via microbial gut alteration [88]. Numerous reviews and meta-analyses have addressed this area; one found a reduction in infant risk of eczema for those under four years with maternal probiotic supplementation during pregnancy and lactation. Another systematic review of 29 trials found that probiotics reduced the risk of eczema when mothers supplemented in the third trimester during breastfeeding and when given to infants [89]. However, no RCT’s have looked at probiotic supplementation exclusively while pregnant.

In summary, the evidence pointing to maternal dietary modulation while pregnant as an effective prevention modality is currently weak, and the current US guidelines do not recommend maternal exclusion of allergens, nor do they recommend any specific food or supplement in the maternal diet while pregnant to prevent food allergy.

### 4.2. Maternal Diet While Breastfeeding

Infants are exposed to antigens through their consumption of mother’s milk. Human milk is known to impart benefits for both maternal and infant health; it contains nutrients necessary for proper growth and development, as well as immunomodulatory components, which contribute to microbiome balance and immunity in infants [90,91]. To date. diet diversity or diet indices while breastfeeding and infant food allergy have not been studied [92]. An RCT demonstrated that omega-3 fatty acid intake during pregnancy and breastfeeding reduced food allergy occurrence, yet this has not been shown exclusively in the post-partum period [87,93]. Additionally, Vitamin D supplementation while breastfeeding has not been associated with reduced food allergy offspring development [87].

The literature on the relationship between maternal consumption of allergenic foods while lactating and infant food allergy are sparse. Several studies have shown that allergens can be transferred through the breastmilk in trace amounts and may offer a protective effect of the subsequent development of food allergy [94,95,96,97]. In addition, data on the influence and protective effects of human milk upon food allergy are inconclusive [90,98]. A recent clinical report from the American Academy of Pediatrics (AAP) states that exclusive breastfeeding for a minimum of three months protects against early wheezing and reduces AD risk. However, findings have been insufficient to suggest whether breastfeeding prevents or delays food allergy [99]. Food allergen avoidance while breastfeeding has not been associated with reduced food allergy outcomes in the lactating infant, and is not recommended in any of the US guidelines [77,78]. The guidelines have also pointed out that allergen avoidance while breastfeeding may lead to nutritional deficiencies. On the other hand, several studies have shown that peanut consumption while breastfeeding may offer a protective effect against peanut sensitization and allergy, yet this strategy has yet to be shown in multiple studies and has not yet been confirmed in RCT’s [96,97]. Similarly, one observational study showed an inverse association between maternal citrus and kiwi fruit consumption while breastfeeding and cow’s milk allergy in offspring [100]. Differing definitions of breastfeeding and poorly defined composition of human milk have affected overall reproducibility.

In summary, maternal dietary modulation while breastfeeding has not been shown to be an effective prevention modality, and the current US guidelines do not recommend maternal exclusion of allergens, nor do they recommend any specific food or supplement in the maternal diet while breastfeeding to prevent food allergy. Additionally, while endorsing exclusive breastfeeding, the guidelines emphasize that this is not a strategy for the primary prevention of food allergy.

### 4.3. Infant Diet

#### 4.3.1. Breastfeeding and Formula Feeding

The World Health Organization (WHO) recommends exclusive breastfeeding for approximately the first 6 months and the continuation of breastfeeding alongside complementary foods for two years and beyond to maximize nutrients and immunological benefits [101,102]. Both breastmilk and infant formula provide all the nutrients that babies need until the age of six months. Depending on the nutritional status of the mother, breast milk continues to provide significant amounts of nutrients past six months; however, key nutrients such as iron, zinc, and vitamin D cannot be met through breast milk consumption or formula alone.

The AAP and the United States Department of Agriculture (USDA) recommend standard infant formula in cases where breastfeeding is not possible or is insufficient [103,104]. Hydrolyzed formulas have been unable to reduce cow’s milk allergy prevalence. However, recent studies indicated that very early introduction of cow’s milk formula and continued intake may prevent cow’s milk allergy, but these studies need further confirmation [105]. While particular brands are not recommended, the type of formula depends on the infant’s specific health needs. The U.S. FDA regulates all commercially sold infant formulas to ensure they meet minimal nutrition and safety requirements [106]. In addition, the FDA requires infant formula to have 30 nutrients, and the AAP and USDA recommend that all infant formula contain iron supplementation. The AAP and USDA recommend that infants not consume cow’s milk as a drink (or any nonhuman milk/formula) until they are 12 months old. In summary, neither breastfeeding, nor specific infant formulas have been shown to prevent food allergies, and are not currently recommended as a means of preventing food allergy.

#### 4.3.2. Supplements

According to a recent systematic review and meta-analysis which included 10 cross-sectional, cohort, and case–control studies, decreased maternal vitamin D levels and infant vitamin D insufficiency both appear to increase the incidence of food allergies, particularly in the second year of life [107]. Risk factors for vitamin D deficiency include exclusively breastfed infants, and/or lack of skin exposure to the sun, dark skin, and medical conditions affecting vitamin D metabolism. Although afternoon sunlight exposure can increase vitamin D stores during the first six months of life, a widely accepted approach to building healthy vitamin D stores in infants who are fed only breast milk or who receive both breast milk and infant formula is through vitamin D supplementation [108]. Recommendations for vitamin D intakes in infancy are available from various organizations throughout the world, and are typically 5 to 10 micrograms daily. Some organizations suggest greater amounts (25 to 30 micrograms daily) as a supplement to exclusive breastfeeding [109]. As infant formulas are fortified with vitamin D, formula-fed babies may not require a supplement. Despite the widespread recommendation for Vitamin D supplementation and the documented association between Vitamin D levels and food allergy, Vitamin D supplementation has not been shown to reduce FA development [87].

#### 4.3.3. Complementary Feeding

The AAP recommends introducing solid foods to infants between four and six months of age. Infants should be fed breast milk or infant formula exclusively for the first six months and until the infant is one year old, the parents should continue breast/formula feeding while gradually introducing solid foods into the infant’s diet. Individual infants may have different readiness cues and rates of progression when starting solids. It is essential for parents and caregivers to tailor the introduction of solid foods based on their unique needs and developmental progress.

During the introduction of solid foods, parents and caregivers should closely monitor infants for any signs of allergic reactions or adverse effects. Any such reactions should be reported promptly to the pediatrician for evaluation and guidance.

Several observational trials have suggested that higher diet diversity in infancy is associated with reduced food sensitization and food allergy, yet this has not been shown in an RCT [110,111,112,113,114,115]. As there is potential food allergy preventive effects, yet no known harm with introducing a wide range of foods, recent guidelines have endorsed feeding infants a diverse diet commensurate with the introduction of complementary foods. In line with this, the AAP now states: “In the past, pediatricians recommended starting one new food every few days, so that you can see if a reaction occurs to that particular food. New research has shown that it is safe to start multiple foods at once.” [116].

The consistency of early solid foods is usually pureed or mashed foods that are easy to swallow. By around 8–10 months, encourage parents to bring their infants texture variety. As the infant becomes more skilled at eating, introducing thicker purees, soft foods, and eventually small, age-appropriate finger foods are encouraged to support their development and oral motor skills. Some parents prefer to use baby-led-weaning, which encourages baby-led-weaning that can be self-fed. However, baby-led-weaning alone may not be adequate, and a modified BLW approach including some spoon feeding may be required to ensure sufficient nutrient intake [117].

The nutritional composition of solid foods is of importance. Ultra-processed foods, typical of the modern Western diet and notably rich in added sugar and chemical additives, are of particular concern. The NOVA classification system, which classifies food according to its level of processing, defines UPF as having undergone a high degree of industrial processing [118]. The AAP recommends that sugar sweetened drinks, the addition of salt to foods, or the consumption of nutrient-poor discretionary foods with high levels of saturated fat, added sugars, and/or added salt should not be included during the transition to solid foods [104]. UPFs have been linked to allergic conditions such as asthma, eczema, and wheezing [119,120]. It has been hypothesized that UPFs can contribute to food allergy development, either due to their poor nutritional composition or as a result of higher levels of food processing [119]. In addition, the low pH of infant foods, particularly infant pouches, has led to concerns regarding an increase in food allergy [121]. As there is currently a dearth of evidence, UPF’s have not yet been addressed in the guidelines for food allergy prevention.

#### 4.3.4. Early Allergen Introduction

The role of cutaneous sensitization and the theory of dual allergen exposure evolved from previous observations that the earlier introduction of peanut into the infant diet in Israel led to lower prevalence of peanut allergy compared to genetically similar cohorts in the UK [122]. The landmark LEAP trial demonstrated that early and regular consumptions of peanut dramatically decreased the incidence of peanut allergy at five years compared to infants avoiding peanut consumption [123]. An extensive body of literature supports this dual allergen exposure hypothesis, and emphasizes the importance of early allergen consumption [122]. Subsequent studies on egg introduction have shown a similar preventive effect, and a recent meta-analysis concluded with moderate certainty that egg introduction between four and six months reduced subsequent egg allergy development [124]. The studies showed that early introduction of well-cooked egg is safer and more effective than raw, pasteurized egg for prevention of egg allergy [125].

**Figure 2:**
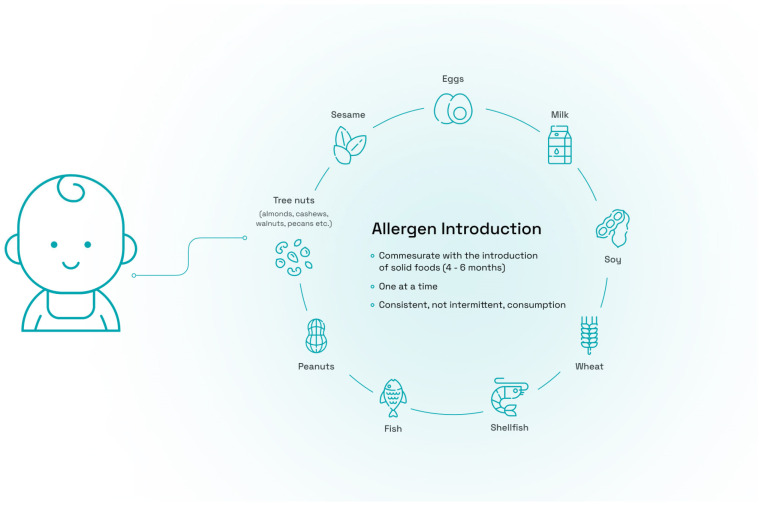
The 2021 Guidance from the AAAAI and ACAAI for the primary prevention of food allergy through nutrition encourages the introduction of peanut and egg at around six months of age, but not before four months of age, to prevent food allergies. Other allergens should be introduced at the same age and after the initial introduction, allergens should be consumed consistently.

Professional societies around the world have endorsed early allergen introduction for the prevention of food allergy (reviewed here) [126]. In the US, this paradigm shift began with the establishment of a coordinating committee representing 26 professional organizations, advocacy groups, and federal agencies, and the convening of an expert panel of representatives in June 2015. Coordinating committee members included the American Academy of Nutrition and Dietetics, the American Academy of Allergy, Asthma and Immunology (AAAAI), the AAP, the American College of Allergy Asthma and Immunology (ACAAI), the American Society for Nutrition, Food Allergy Research and Education (FARE), the National Institute of Allergy and Infectious Diseases, the North American Society for Pediatric Gastroenterology, Hepatology and Nutrition, and the World Allergy Association. The committee published guidelines for the prevention of peanut allergy [77], and endorsed the early introduction of peanuts in high risk infants. In 2019, the AAP issued updated guidelines which stated that “there is no evidence that delaying the introduction of allergenic foods, including peanuts, eggs, and fish, beyond 4 to 6 months prevents atopic disease. There is now evidence that early introduction of peanuts may prevent peanut allergy.” [78]. The Dietary Guidelines Advisory committee of the USDA stated that the evidence indicates that introducing peanut and egg in the first year of life (after age four months) may reduce the risk of food allergy to peanuts and eggs in 2020 [127]. In 2021, the AAAAI and the ACAAI extended the guidelines to additional allergens and suggested that both peanut and egg should be introduced around six months of age, but not before four months of age. They also suggested that other allergens be introduced at the same time (Figure 2).

## 5. Conclusions

Food allergy guidelines have changed over the past two decades. Our understanding of risk factors and their contribution towards the development of food allergy is lacking, which makes food allergy prevention guidelines relevant to all families. Food allergy prevention guidelines state that there is no evidence to advise pregnant and breastfeeding women to avoid food allergens. Breastfeeding and use of hydrolyzed formulas are not associated with reduced prevalence of food allergies. Breastfeeding is, however, recommended for all mother-infant dyads due to a range of other health benefits. Peanut and cooked egg should be introduced as soon as solid food introduction commences. Introduction of other food allergens should not be delayed. Once an allergen is introduced, frequent and continued intake is required to maintain tolerance. Alongside food allergen intake, the overall infant diet should also be addressed to provided optimum diet diversity and support a diverse gut microbiome. There is currently no evidence that the use of any supplement prevents food allergy. Dietitians play a crucial role in providing practical information regarding early life feeding, including all aspects of food allergen intake, overall diet, supplement use, and formula intake, if required, to families, and their role in food allergy prevention should be encouraged. However, to help stem the tide of food allergies, dietitians may need to be trained in food allergy risk factors and their prevention. The particular role of the dietitian includes a range of different skills and knowledge (See Sidebar 2).

Sidebar 1. *Guidelines on Feeding with Formula (Adapted from the USDA & AAP)*

First Days:

- In total, 1 to 2 ounces of infant formula every 2 to 3 h in the first days of life.
- Give more if showing signs of hunger.
- Most infant formula-fed newborns will feed 8 to 12 times in 24 h.

First Weeks and Months:

- Feed every 3 to 4 h.
- Babies will generally take what they need at each feeding and stop eating when they are full.
- In total, 3 to 4 ounces (90 to 120 mL) per feed and 32 ounces per day.
- If an infant sleeps longer than 4 to 5 h during the first few weeks after birth and starts missing feedings, parents should wake them up and offer a bottle.
- Provide Vitamin D Supplementation if the infant drinks less than 32 ounces of infant formula a day,
- By 6 months: Your baby will consume 6 to 8 ounces (180–240 mL) at each of 4 or 5 feedings in 24 h.

Measurements:
1 ounce = 30 cc (cubic centimeters) = 30 mL (milliliters)
8 fluid ounces = 1 cup
32 fluid ounces = 1 quart

Scoop of powder to every 2 fluid ounces of water.
In total, 2 fluid ounces of concentrate with 2 fluid ounces of water.

Sidebar 2. *Dietitian’s Role in Food Allergy Prevention*

- Support breastfeeding.
- Nutritional guidance to the breast-feeding parent.
- Advice on suitable infant formula (if needed).
- Guidance on responsive feeding styles.
- Advice on nutritionally appropriate foods.
- Advice on suitable textures and texture progression.
- Introduction of food allergens in terms, including:
  ○ amount;
  ○ type;
  ○ frequency.
- Monitoring growth and nutritional status.
- Incorporating food allergens into the usual family diet.
- Overcoming food aversions.

## List of Abbreviations

AAAAI	American Academy of Allergy, Asthma and Immunology
AAP	American Academy of Pediatrics
ACAAI	American College of Allergy, Asthma, and Immunology
AD	Atopic Dermatitis
DII	Dietary Inflammation Index
FA	Food Allergy
FARE	Food Allergy Research and Education
FDA	Food and Drug Administration
FLG	Filaggrin
HEI	Healthy Eating Index
IgE	Immunoglobin E
IL	Interleukin
LEAP	Learning Early About Peanuts
RCT	Randomized Controlled Trial
sIgE	Specific Immunoglobin E
US	United States
USDA	United States Department of Agriculture
VD	Vaginal Delivery
WHO	World Health Organization
